# Artificial Intelligence (AI) in rheumatology: a comparative evaluation of the ChatGPT and DeepSeek application

**DOI:** 10.1186/s41927-026-00618-y

**Published:** 2026-02-12

**Authors:** Maria Polyzou, Xenofon Baraliakos

**Affiliations:** 1 Ruhr University Bochum, Rheumazentrum Ruhrgebiet, Herne, Germany; 2 Department of Pathophysiology, School of Medicine, National and Kapodistrian University of Athens, Laiko General Hospital, Athens, Greece

**Keywords:** Artificial Intelligence (AI), Large Language Models (LLMs), Rheumatology, Ankylosing spondylitis (axSpA), Psoriatic arthritis (PsA), ChatGPT, DeepSeek

## Abstract

**Background:**

The continuous increase in Artificial Intelligence (AI) applications in various areas of human life has brought about great changes in many sciences, among which is the health sector. ChatGPT and DeepSeek belong to the category of Large Language Models (LLMs) developed by Artificial Intelligence (AI) using supervised and reinforcement learning techniques. The aim of this article is to evaluate the accuracy and consistency of ChatGPT and DeepSeek models in the diagnosis and treatment of two rheumatologic diseases, ankylosing spondylitis (axSpA) and psoriatic arthritis (PsA).

**Methods:**

Both ChatGPT and the DeepSeek chat system have revolutionized information retrieval capabilities and are two of the fastest growing platforms. They are effective tools that produce text responses to human data with high accuracy, accessibility, and low cost, but their use has raised many questions about their reliability. The evaluation carried out in this article is done by comparing the responses obtained from the two models with the results of clinical findings in axSpA and PsA, using four statistical tests. Specifically, the comparison of the responses with the clinical data obtained from 116 patients, who were hospitalized for rheumatological diseases at the Rheumazentrum Ruhrgebiet in Herne, was carried out by calculating the differences in the mean values of the estimates, the Cohen Kappa coefficient, the Fleiss’ Kappa coefficient and the confidence level corresponding to the differences in the mean values, as well as from the calculation of certain other statistical indicators.

**Results:**

Regarding the comparison of the mean values, there are results in which their coincidence for the three cases examined is very good, in other cases it is satisfactory, while in the rest there are large differences. Regarding the results of the calculation of the Cohen’s Kappa coefficient, no agreement is indicated between the clinical results and the answers of ChatGPT and DeepSeek and specifically the GPT-5 and DeepSeek-R1 models. The results of the calculations of the Fleiss’ Kappa coefficient showed that also, no satisfactory agreement was found in the values ​​of the clinical data, with the answers of ChatGPT and DeepSeek. The results obtained from the calculation of certain other statistical indicators, as well as the probabilities corresponding to the differences between the mean values ​​of the results obtained from the two models and the clinical findings, are similar.

**Conclusions:**

The final results and quantitative assessments of the analysis showed that the responses of the ChatGPT and DeepSeek models have moderate validity, reliability and utility in providing information to patients with axSpA and PsA. Therefore, the use of the information obtained from these models should be done after relevant evaluation and validation by doctors and cross-checking the recommendations with updated clinical guidelines.

**Clinical trial number:**

Not applicable.

**Supplementary Information:**

The online version contains supplementary material available at 10.1186/s41927-026-00618-y.

## Background

Artificial intelligence (AI) includes patterns of learning by leveraging prior data, natural language understanding, perception, reasoning and problem-solving [21]. The applications of AI in various areas of human life, including the health sector, have been increasing exponentially [5, 6]. AI has brought about significant changes in health science research and multiple applications of AI are gradually being utilized in the field of rheumatology. These changes affect medical diagnosis and the ways of treating patients [28]. The use of AI to support decision-making has helped improve clinical expertise, particularly in fields that require complex data interpretation, such as radiology, genomics and rheumatology [10].

The ability of AI to process large data sets and recognize patterns beyond human ability has paved the way for precision medicine [11]. In particular, the use of AI in rheumatology, which includes multisystem pathologies, overlapping symptoms, and complex inflammatory pathways, is particularly important [28]. There are many applications of AI in rheumatology. The use of machine learning (ML) and deep learning (DL) algorithms in diagnostic imaging is particularly noteworthy [8]. Understanding autoimmune diseases can be facilitated by leveraging AI through biomarker discovery and genomic analysis [11]. Also, remote disease monitoring and patient self-management can be greatly facilitated by improvements in digital health technologies and wearable devices. New digital tools can help implement telemedicine and home care, through real-time disease monitoring and automated flare-up detection [10].

The use of AI in predictive analytics is of particular interest in rheumatology. Predictive AI uses big data analytics and deep learning to examine historical data, patterns, and trends. The reliability of predictions depends on the amount of data provided to machine learning algorithms. In rheumatology, predictive AI involves training machine learning models to predict disease trajectories. Leveraging predictive analytics gained through the use of AI allows rheumatologists to manage diseases more effectively, focusing on preventive intervention strategies. Thus, minimizing disease-related complications and optimizing patient recovery is achieved [28].

In recent years, a new type of AI model designed to understand natural language as well as generate it on a large scale, namely Large Language Models (LLMs). In medicine, AI applications, including patient symptom checkers, represent an area of interest and could facilitate patient triage and accelerate diagnosis [22, 23]. A LLM is a type of AI algorithm that uses various deep learning techniques, such as neural networks and big data, to produce text similar to human speech. In addition, LLMs can perform a large number of natural language processing (NLP) tasks, including text generation, sentiment analysis, and text translation.

LLMs follow self-supervised learning approaches, which show remarkable performance on all kinds of natural language tasks [16, 17]. It is noted that each LLM is trained on huge sets of data or parameters— hence the name “large” - often ranging from millions to billions. For example, the number of parameters used by GPT-4 is about 1 trillion. In general, the introduction of LLMs such as ChatGPT and DeepSeek, has raised expectations for their use in medicine as an auxiliary diagnostic tool [22, 26, 27].

LLMs, with well-known examples being ChatGPT and DeepSeek models, can be powerful tools with potential applications in many fields, including rheumatology [9]. The applications of LLMs rely on their ability to understand complex texts and generate new text. For example, OpenAI’s GPT-5 has become ubiquitous in many areas, while DeepSeek-R1, a model acquired in 2025, aspires to compete with ChatGPT by offering increased performance through its Mixture-of-Experts (MoE) model [27].

Rheumatology, as a medical specialty dealing with diseases of the musculoskeletal system and connective tissue, is characterized by the complexity of diagnoses, the management of chronic diseases, and the need for continuous updating on developments in research and therapeutic approaches. These diagnoses can be accelerated and improved through the analysis of large amounts of data and the recognition of patterns that may not be immediately apparent. In this context, LLMs can offer significant assistance to physicians and patients [17, 22, 31].

As mentioned above, LLMs having been trained on large amounts of data, have the ability to combine symptoms, laboratory results, and images, in conjunction with other AI tools, significantly aiding in the successful diagnosis of a rheumatologic condition. For example, if a patient’s laboratory tests show elevated levels of antibodies, ChatGPT can identify patterns that are often associated with specific rheumatic diseases, such as systemic lupus erythematosus or rheumatoid arthritis, providing doctors with important information about the treatment to be applied [17].

The question that concerns the medical community is related to the capabilities and reliability provided by the use of the AI computational tools ChatGPT and DeepSeek. This article attempts to contribute to the answer to this question. Specifically, the evaluation of the potential uses in rheumatology and the reliability provided by the ChatGPT and DeepSeek will be done. The evaluation will be done by statistically testing the results obtained using the GPT-5 and DeepSeek-R1 application through answers to specific questions. The answers and results obtained will be compared with corresponding clinical findings of patients at the Rheumazentrum Ruhrgebiet in Herne, University Hospital of Bochum, Germany. The examination will be done on patients suffering from two rheumatological diseases, ankylosing spondylitis (axSpA) and psoriatic arthritis (PA).

## Evaluation of LLMs application in medical sector: a narrative of literature review

LLMs use Genetic AI, which is a field of research with the potential to create new content, such as text generation. The ability to produce new content creates the conditions for many applications in the healthcare sector [1, 36]. Genetic AI uses the principles of Machine Learning, which allows machines to gain knowledge from data [30]. Unlike conventional machine learning models that extract patterns and make decisions based on them, genetic AI is trained using data and creates new variants that reflect the characteristics of the original data [4]. The rapid growth in AI has immensely changed NLP, with two prevalent LLMs in the form of ChatGPT and DeepSeek.

In recent years, the use of LLMs has been increasing due to their remarkable performance in various practical applications, leading to the accelerated development of a large number of different models [35]. LLMs have generated great excitement for their potential applications in many sciences [13]. Understanding the accuracy and reliability of the medical “knowledge” stored in the parameters of these models and developing confidence measures in their responses is of particular importance. The probability that an LLM response is accurate is critical for the safe and effective use of these tools in any science, especially in the health sciences, where the cost of a wrong choice can be very high.

The most well-known LLMs are GPT, DeepSeek, BERT, LLaMA, PaLM, Gemini, Mistral, Claude 4 and Grok 4. However, the use of these LLMs cannot be generalized to complex tasks due to inherent training biases, model size limitations and the quality or diversity of the datasets before training [25]. Recent developments in LLMs, such as improvements through reinforced retraining, have enhanced reasoning abilities, as demonstrated by models such as OpenAI-o1 and DeepSeek-R1 [20].

The use of LLMs, such as ChatGPT and DeepSeek, includes a wide range of capabilities, which are constantly improving, making LLMs indispensable in many scientific fields. The most important uses can be mentioned as follows: (a) creating text of various formats, (b) answering questions with accuracy and detail, using the knowledge they have acquired from their training, (c) translating text from one language to another with high accuracy and using complex language structures, (d) summarizing text into smaller and more concise versions, (e) generating code, (f) sentiment analysis etc.

LLMs have the potential to play a critical role across the entire research spectrum of the health sector. Thus, they can help researchers identify key discoveries and therapeutic options by quickly scanning huge amounts of medical literature and clinical data. Research reports, summaries, and even compelling research papers can be written with their ability to produce coherent and contextually relevant content [24].

However, although LLMs produce text and responses that appear plausible, their reliability is not guaranteed. The answers generated by LLMs are not based on the accuracy of the information in the training data, but are generated as the probabilistically most convincing texts. Therefore, these answers require verification, because LLMs continue to face challenges, particularly in reliably sourcing information [33]. In other words, providing reliable information is a key success factor for the use of LLMs, while producing inaccurate results is commonly referred to as “hallucinations”. This raises serious questions about the reliability and use of LLMs in sensitive areas such as healthcare. Incorrect medical information may put patients at risk when diagnosis and treatment recommendations are based solely on them, without evaluation by healthcare professionals [33].

Despite these improvements, the abilities of LLMs are mainly evaluated in areas such as mathematical problem solving and code generation, without answering the question of the generalization of reasoning skills to complex real-world scenarios. Criticisms of LLMs for lacking real “understanding” and “reasoning” are common, being seen as mere auto-completers [38].

Therefore, the legitimate question arises about how to verify the reliability of the results provided by LLMs, to ensure their safe use in the healthcare sector. Assessing the reliability of results derived from the application of LLMs in healthcare is a critical and complex endeavor due to the sensitive nature of medical information. Despite the fact that the emergence of LLMs and their widespread use occurred in 2022, many studies have appeared in the international literature regarding how to assess the reliability of LLMs, especially for those referred to in the healthcare sector.

A common practice for assessing the effectiveness of LLMs is to use evaluation criteria such as MMLU, ARC-C, PIQA and HellaSwag. This practice involves formulating questions in an initial form and then checking the variability of responses to different reformulations of the same question [24, 34]. However, meaningfully evaluating LLMs in their use for solving real-world problems comes with a distinct set of challenges, and reservations are expressed about the adequacy of synthetic benchmarks and de-facto measures often used in the literature [35].

In a general view, the methods for assessing the reliability of LLMs results are divided into three categories:

- The first category includes methods that compare LLMs results with clinical research results and Gold-Standard data. In other words, LLMs results are compared with established medical databases, clinical guidelines and peer-reviewed literature [22]. Often, the evaluation is accompanied by appropriate statistical analysis to check the reliability of the results and calculate various statistical metrics or measures [3, 12]. This helps to quantify the accuracy and validity of the information produced by the models.
- The second category includes methods in which LLMs results are evaluated by expert health professionals with appropriate review and validation of these results, whose opinions serve as a reference standard [3, 33]. This means that clinicians and medical experts review the content generated by LLMs for accuracy, clinical relevance, and appropriateness. This assessment ensures that the information provided by LLMs is aligned with current medical knowledge and best practices. In several studies, the generated answers were rated for accuracy by independent health consultants using a five-point (0 = completely inaccurate to 4 = completely accurate) or ten-point Likert scale [41, 42].
- The third category includes the Clinical Decision Support Evaluation, in which an assessment of how well the LLMs support clinical decision making is performed. In this way, the LLMs’ ability to provide accurate and relevant information for diagnosis, treatment planning, and patient management is assessed [18, 19]. This may include simulated clinical scenarios or retrospective analysis of real-world data [30].

The LLMs used and evaluated in this article are ChatGPT and DeepSeek. Various criteria for selecting LLMs are proposed in the literature, depending on their use [2, 38]. The criteria for selecting these LLMs in this article were their accessibility and practicality for use, as well as the very high percentage of their use for medical applications that appears in the literature. It is noted that the main objective of the article is to create a computational evaluation procedure, of the two LLMs, through the proposed methods and indicators, which is suitable for use in medical sciences and can obviously be applied to each of the LLMs.

## Evaluation of the ChatGPT and DeepSeek application in rheumatology: methods and results

In this section, the potential use of the GPT-5 and DeepSeek-R1 models in rheumatology is evaluated, while their reliability is estimated with appropriate statistical analysis. The evaluation will be done by utilizing the answers received from the two AI models to questions given to them. Specifically, 28 questions were given to the two models, both for two rheumatological diseases, axial spondylitis and psoriatic arthritis, while the corresponding answers were also received. The quantitative estimates obtained from the two models for the rheumatological diseases are compared with the clinical outcomes and relevant measurements, as obtained from a tertiary university hospital specialized in these diseases. The clinical data used in the study were obtained from 1-3-2021 to 31-8-2021 and concerned patients hospitalized at the Rheumazentrum Ruhrgebiet in Herne, at the University Hospital Bochum, Germany. The total number of patients was 116, their age ranged from 35 to 70 years and of these 68 were women and 48 were men.

The questions that were given to the two models GPT-5 and DeepSeek-R1 are shown in Table A-1, in Appendix. The results of the answers obtained from the GPT-5 model, are shown in Table A-2, as well as the results of the answers obtained from the DeepSeek model are shown in Table A-3. Finally, the values ​​of the measurements taken from patients and corresponding to the questions given in the two models are shown in Table A-2. Specifically, the third column of Table A-2 shows the percentage of patients $\:\widehat{\boldsymbol{p}}$ in the total number of patients in the survey, who have the specific characteristic corresponding to the question in the same row.

It is known from statistics that, when, from the available information of a random sample, we seek to estimate the proportion of P individuals in the population who have a given property or characteristic, then we follow the methodology of estimating proportions [7, 40]. Thus, if in the sample of size n the individuals who have the characteristic we are examining are x, then the proportion in the sample will be:

1 $$\:P=\widehat{p}=\frac{x}{n}$$

The sample proportion $\:\widehat{p}$ will follow the binomial distribution and the following relationships apply [6, 28]:

2 $$\eqalign{& E({x \over n}) = E(\hat p) = P{\mkern 1mu} {\rm K}\alpha \iota {\mkern 1mu} {\rm{var}}({x \over n}) = {\rm{var}}{\mkern 1mu} (\hat p) \cr & \Rightarrow \sigma _p^2 = {{p(1 - p)} \over n} \cr} $$

If the sample size is large, then the distribution of $\:\widehat{\boldsymbol{p}}$ approaches the normal distribution [6]. The statistical tests that will follow includes:

### Comparison of the differences between the mean values ​​of the estimates

**Fig. 1:**
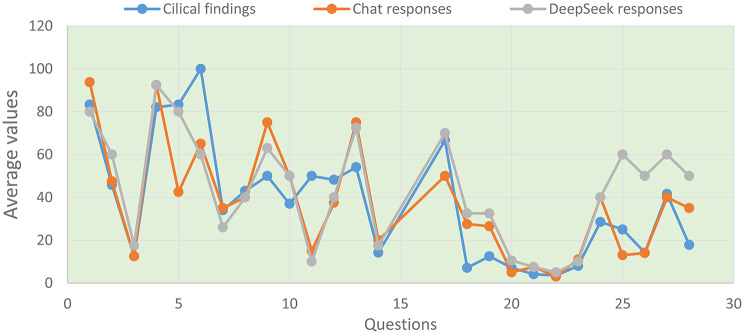
Comparison of mean values ​​of clinical findings and responses of AI models. The Figure shows the average values ​​of the clinical measurements and the results obtained from the responses of the GPT-5 and DeepSeek-R1 models in order to check their degree of coincidence

In the first statistic test, the mean values ​​of the results obtained from the clinical measurements, as well as the results obtained from the responses of the GPT-5 and DeepSeek-R1 models, are compared in order to check the degree of their coincidence. The diagrammatic representation of the mean values ​​for the three cases examined, which is shown in Fig. 1, helps to better illustrate their differences. A macroscopic view of Fig. 1 leads us to the conclusion that there are results in which the coincidence of the mean values ​​for the three cases examined is very good, in some other cases it is satisfactory, while in the rest there are large differences in the mean values.

### Cohen Kappa coefficient estimation

The second statistic test includes a measurement of agreement between the mean values of the three categories by estimating the Cohen’s Kappa statistic. We note that the main objective of the analysis that follows in the next two paragraphs is to calculate the degree of agreement of the mean values ​​of the three categories (i.e. clinical findings, GPT-5 responses, and DeepSeek-R1 responses). To perform this analysis, in this paragraph we calculate the Cohen Kappa coefficient.

The Cohen’s Kappa statistic is frequently used to test interrater reliability. In other words, it is typically utilized to assess the level of agreement between two raters, when there are two categories [4, 24, 25]. Despite the fact that the Cohen’s Kappa statistic is typically utilized to assess the level of agreement when there are two categories or for unordered categorical variables with three or more categories, we believe that calculating these indicators will provide a general picture of the reliability of the two LLMs [25]. Kappa and percent agreement are compared, and levels for both Kappa and percent agreement that should be demanded in healthcare studies are suggested [29, 37]. Given that in our study there are three categories of values, the Kappa coefficient will be calculated for three combinations, while the results of the calculations made using SPSS (version 29) are shown in Table 1.

**Table 1:** Cohen’s Kappa coefficient Estimation for the mean values of clinical findings and responses of AI models

Measure of Agreement between:		Value	Asymptotic Standard Error^a^	Approximate T^b^	Approximate Significance
Clinical findings and ChatGPT responses	Kappa	0,03	0,035	1.719	0,086
Clinical outcomes and DeepASheek responses	Kappa	-0,009	0,007	-0,532	0,595
ChatGPT and DeepSeek responses	Kappa	0,170	0,071	5.688	< 0,001

N of valid cases = 26, (a) Not assuming the null hypothesis. (b) Using the asymptotic standard error assuming the null hypothesis. - The Kappa coefficient is calculated using SPSS (version 29) for the three study combinations

The value for Cohen’s Kappa coefficient always ranges between − 1 and 1. If this value is < 0, it indicates that there is no agreement between the two raters, while it is equal to 1, it indicates perfect agreement between the two raters. More specifically, Cohen suggested the Kappa result be interpreted as follows: values ≤ 0 as indicating no agreement and 0.01–0.20 as none to slight, 0.21–0.40 as fair, 0.41– 0.60 as moderate, 0.61–0.80 as substantial, and 0.81–1.00 as almost perfect agreement [4].

Evaluating the results of the Cohen’s Kappa coefficient calculations shown in Table 1 for the three categories of values, no agreement is indicated for Clinical findings and GPT-5 responses, as well as for Clinical outcomes and DeepSheek-R1 responses. Also, based on the corresponding values ​​of the p-value of the null hypothesis test, the degree of agreement for these categories is not statistically significant. On the contrary, for the values ​​of the categories GPT-5 and DeepSheek-R1 responses the degree of agreement is statistically significant, but it is very small.

### Fleiss’ Kappa coefficient estimation

The third statistic test includes a measurement of agreement between the mean values of the three categories by estimating the Fleiss Kappa statistic. The aforementioned Cohen Kappa statistic is applicable to pairs of raters, while the Fleiss Kappa is defined for measuring the agreement among more than two raters [37]. Like Cohen’s Kappa, Fleiss’ Kappa index is commonly used to assess the level of agreement on categorical variables. In this case, we believe that calculating the index will provide a general picture of the reliability of the two LLMs.

The results of the Fleiss’ Kappa coefficient calculations are shown in Table 2 and are similar to the results in Table 1. No agreement in the values ​​of the clinical outcomes was found, with the responses of GPT-5 and DeepSheek-R1. Also, based on the corresponding values ​​of the p-value of the null hypothesis test, the degree of agreement for these categories is not statistically significant. It is noted that the results of the answers to questions 15 and 16 are not included in the calculations of Cohen and Fleiss’s Kappa coefficients, as the answers of the GPT-5 and DeepSeek-R1 models are not clear, as shown in Tables A-2 and A-3 in the Appendix.

**Table 2:** Fleiss’ Kappa coefficient Estimation for the mean values of clinical findings and responses of AI models

	Kappa	Asymptotic Standard Error^a^	Z	Asymptotic Significance	Asymptotic 95% Confidence Interval	
					Lower Bound	Upper Bound
Measure of overall Agreement	0,047	0,020	0,019	0.019	0.008	0.086

N of Valid Cases = 26, Sample data contains 3 raters. This statistical test involves measuring the agreement between the means of the three categories by estimating the Fleiss Kappa coefficient

### Confidence level corresponding to the differences of the mean values estimation

The fourth statistic test includes the estimation of confidence level corresponding to the differences d of the mean values ​​of the clinical outcomes with the mean values ​​of the responses given by the GPT-5 and DeepSheek-R1 models. Thus, if m is each mean value corresponding to the clinical outcomes and m_i_ (i = 1, 2) each mean value corresponding to the responses of the GPT-5 and DeepSheek-R1 models respectively, then it will be: d_i_=m-m_i_. The precision of the confidence interval for a population parameter is inversely proportional to its range. As the width of the interval increases, the estimation accuracy decreases and therefore the information content of the interval for the estimated parameter decreases.

To calculate the width of the confidence interval as a function of the other parameters, assuming that the values ​​of clinical findings and responses of the GPT-5 and DeepSeek-R1 models follow the normal distribution, we distinguish the following cases:

#### For the population mean m

For a confidence level of 1-α, we estimate the following interval for the mean value:

3 $$(\bar x - {{\rm{Z}}_{\alpha /2}}{s \over {\sqrt n }},\bar x + {{\rm{Z}}_{\alpha /2}}{s \over {\sqrt n }})$$

The width of the interval d is equal to:

4 $$d = 2{{\rm{Z}}_{1 - \alpha /2}}{s \over {\sqrt n }} \Rightarrow {{\rm{Z}}_{1 - \alpha /2}} = {{d\sqrt n } \over {2s}}$$

#### For the population proportion p

For a confidence level of 1-a, we estimate the following interval for the p-value:

5 $$(\hat p - {{\rm{Z}}_{1 - \alpha /2}}\sqrt {{{\hat p(1 - \hat p)} \over n}} ,\,\hat p + {{\rm{Z}}_{1 - \alpha \>/2}}\sqrt {{{\hat p(1 - \hat p)} \over n}}$$

The width of the interval d is equal to:

6 $$\eqalign{ & d = 2{{\rm{Z}}_{1 - \alpha \>/2}}\sqrt {{{\hat p(1 - \hat p)} \over n}} \cr & \Rightarrow \>{{\rm{Z}}_{1 - \alpha \>/2}} = {d \over {2\sqrt {{{\hat p(1 - \hat p)} \over n}} }} \cr}$$

For the values of Z_1−α/2_, which result from the use of Eqs. (4) and (6), it is possible to calculate the corresponding probabilities from the relevant tables of the normal distribution. Equation (4) will be used to calculate the probabilities corresponding to questions (7) and (8), while Eq. (6) will be used to calculate the probabilities of the remaining questions in Table A-1. Using the clinical data and the data derived from the use of the GPT-5 and DeepSeek-R1 models, the values ​​of Z_1−α/2_ are calculated and subsequently the corresponding probabilities. It is obvious that the higher the probability values, the higher the values ​​of the differences d_i_=m-m_i_ and therefore the lower the coincidence of the clinical results with the responses of the GPT-5 and DeepSheek-R1 models.

**Fig. 2:**
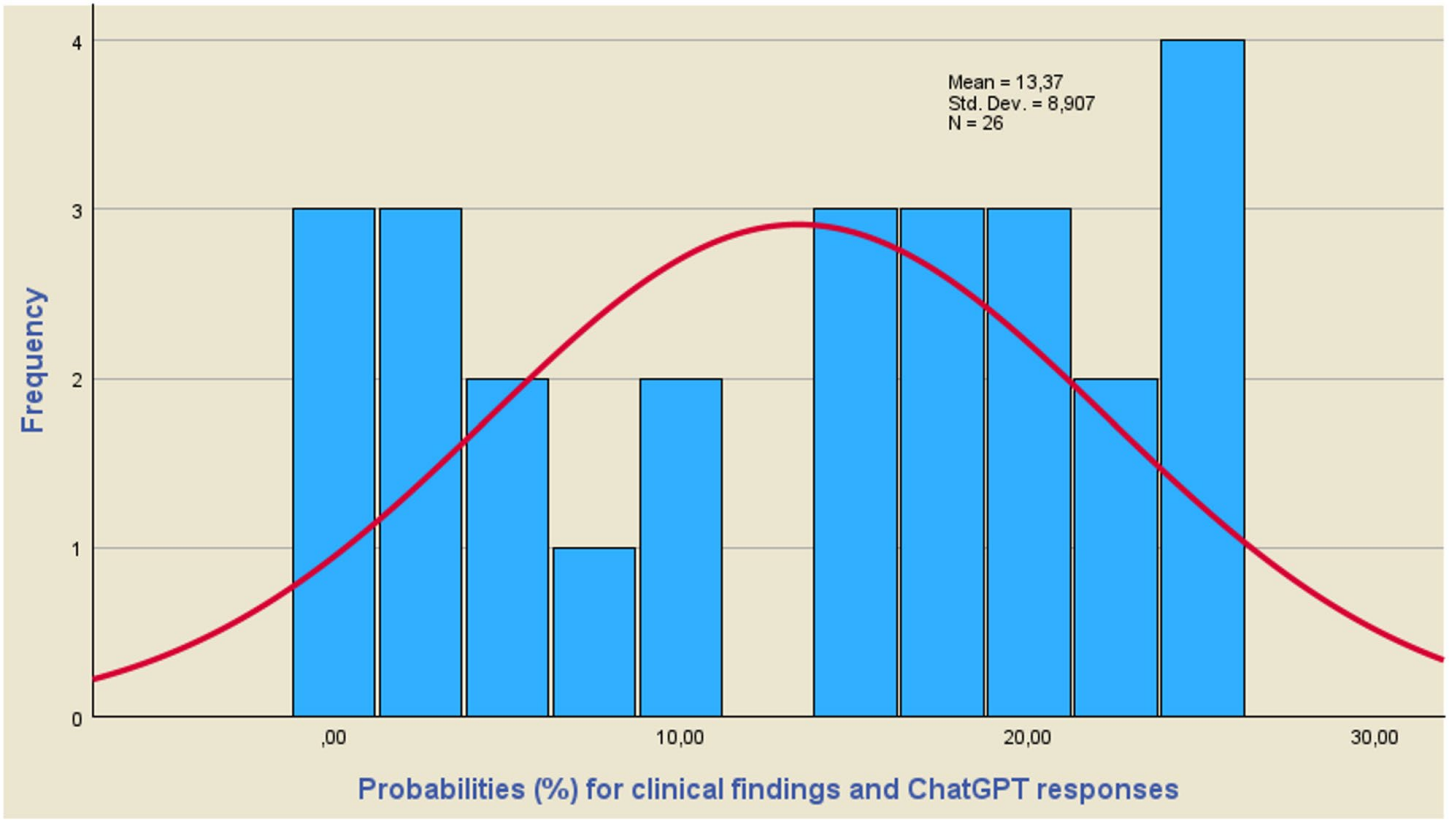
Probabilities (%) corresponding to the differences d of the mean values of Clinical findings and ChatGPT responses. The calculated probability values ​​do not follow a normal distribution satisfactorily

**Fig. 3:**
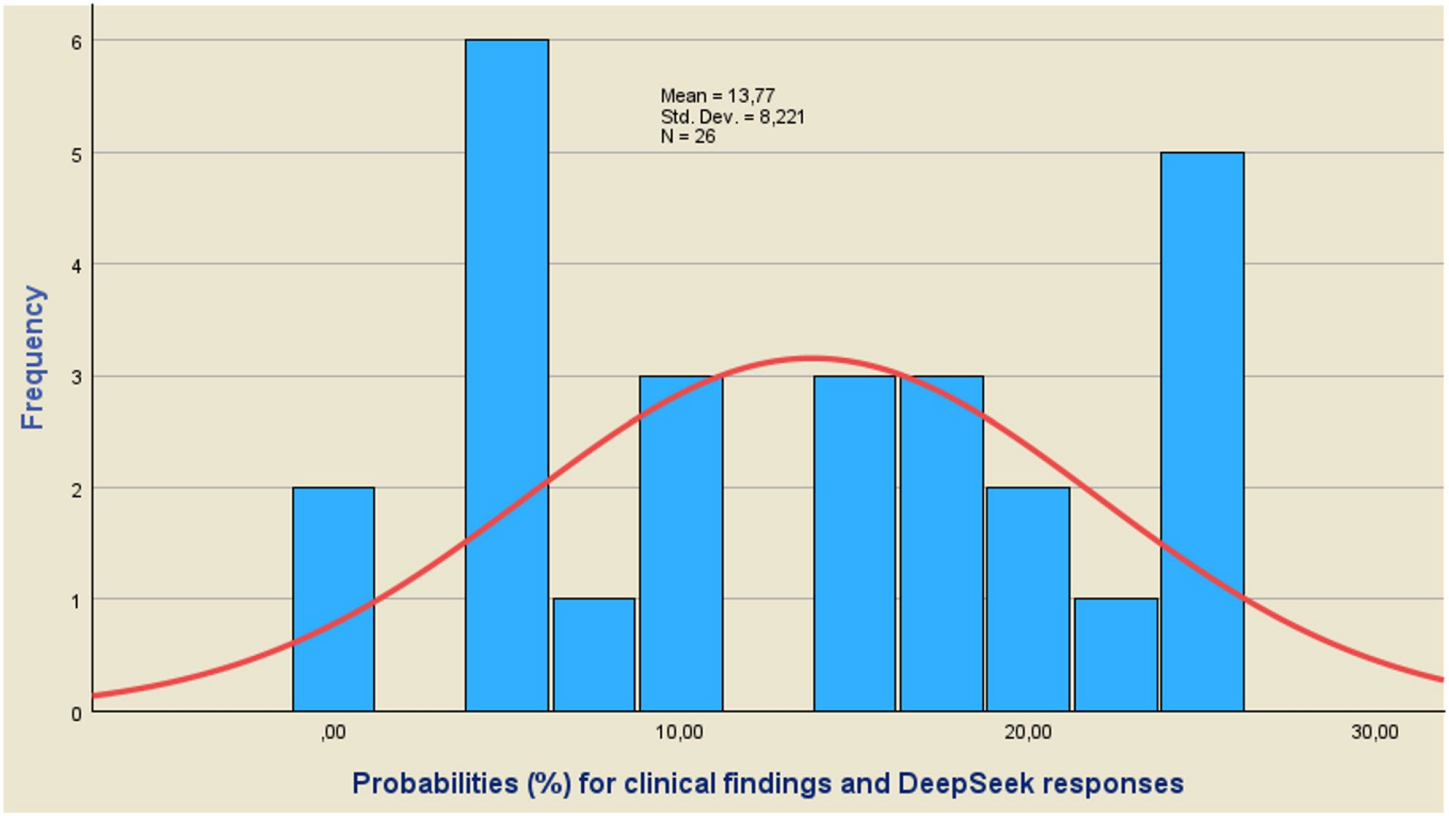
Probabilities (%) corresponding to the differences d of the mean values of Clinical findings and DeepSeek responses. The calculated probability values ​​do not follow a normal distribution satisfactorily

**Fig. 4:**
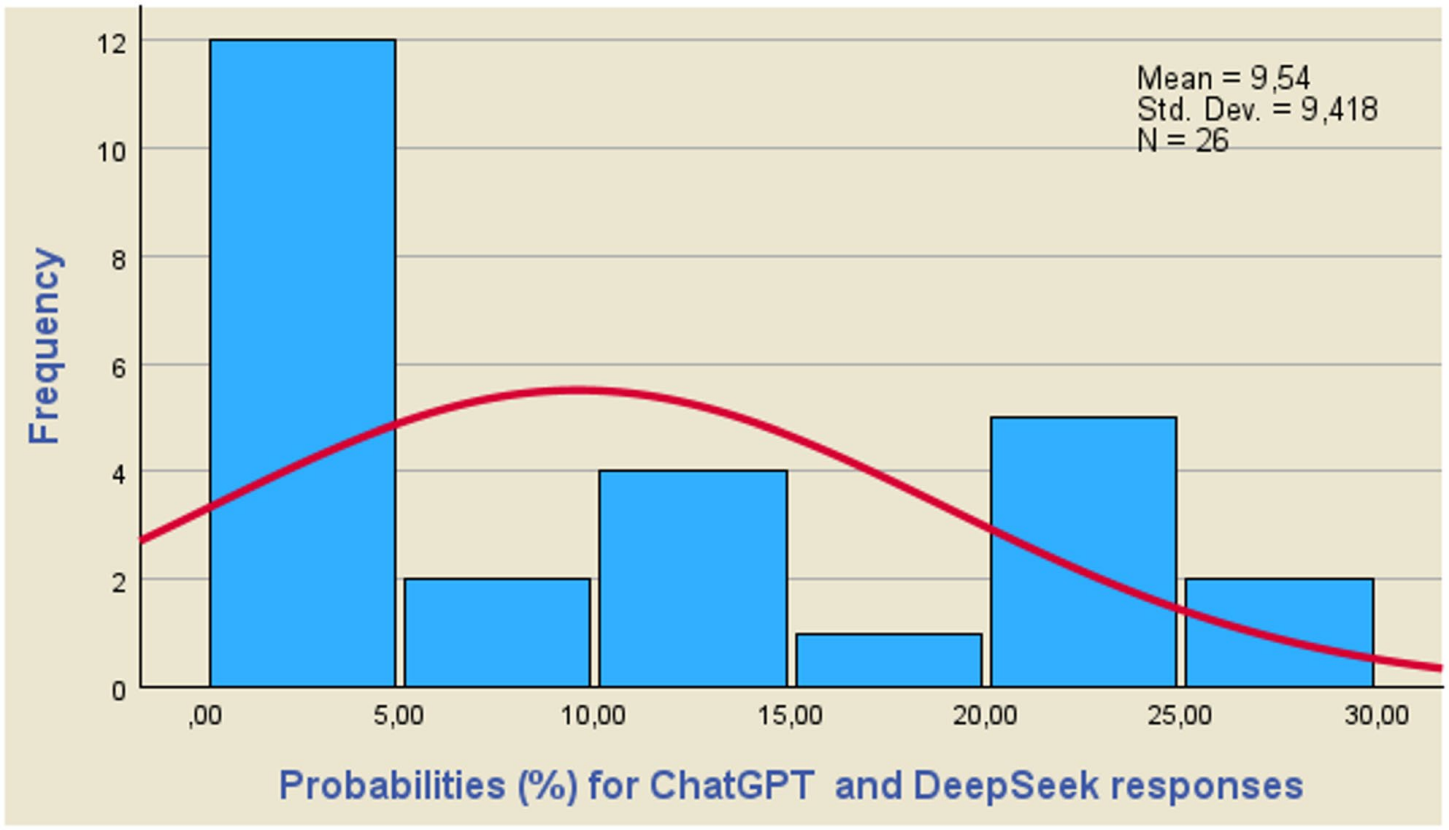
Probabilities (%) corresponding to the differences d of the mean values of GPT-5 and DeepSeek-R1 responses. The calculated probability values ​​do not follow a normal distribution satisfactorily

Using the results obtained from these calculations, the histograms of Figs. 2, 3 and 4 are constructed, while the parameters of the Tests of Normality of the Kolmogorov-Smirnov (K-S) and Shapiro-Wilk (S-W) tests are calculated and shown in Table 3. A macroscopic view of Figs. 2, 3 and 4 leads to the conclusion that the values ​​of the probabilities corresponding to the differences d = m-mi do not guarantee a satisfactory coincidence of the predictions obtained using the GPT-5 and DeepSeek-R1 models with the clinical results.

**Table 3:** Parameters of tests of normality

Tests of Normality						
	Kolmogorov-Smirnov^a^			Shapiro-Wilk		
	Statistic	df	Sig.	Statistic	df	Sig.
Clinical findings-ChatGpt responses	0,155	26	0,111	0,898	26	0,014
Clinical findings - DeepSeek responses	0,145	26	0,166	0,920	26	0,004
ChatGPT - DeepSeek responses	0,218	26	0,003	0,837	26	< 0,001

a. Lilliefors Significance Correction. - The Table includes the parameters of the Kolmogorov-Smirnov (K-S) and Shapiro-Wilk (S-W) Normality Tests

The calculated probability values ​​do not follow a normal distribution satisfactorily, while the mean values ​​for the clinical findings, the responses of the GPT-5 and DeepSeek-R1 models are 13.37, 13.77 and 9.54 for their combinations. The conclusion that the calculated probability values ​​do not follow a normal distribution is also confirmed by Table 3, where the parameters of tests of normality are displayed. As a rule of thumb, we conclude that a variable is not normally distributed if “Sig.“<0.05. Thus, both the Kolmogorov-Smirnov test and the Shapiro-Wilk test suggest that only the results concerning the combination of GPT-5 and DeepSeek-R1 responses follow a normal distribution.

**Fig. 5:**
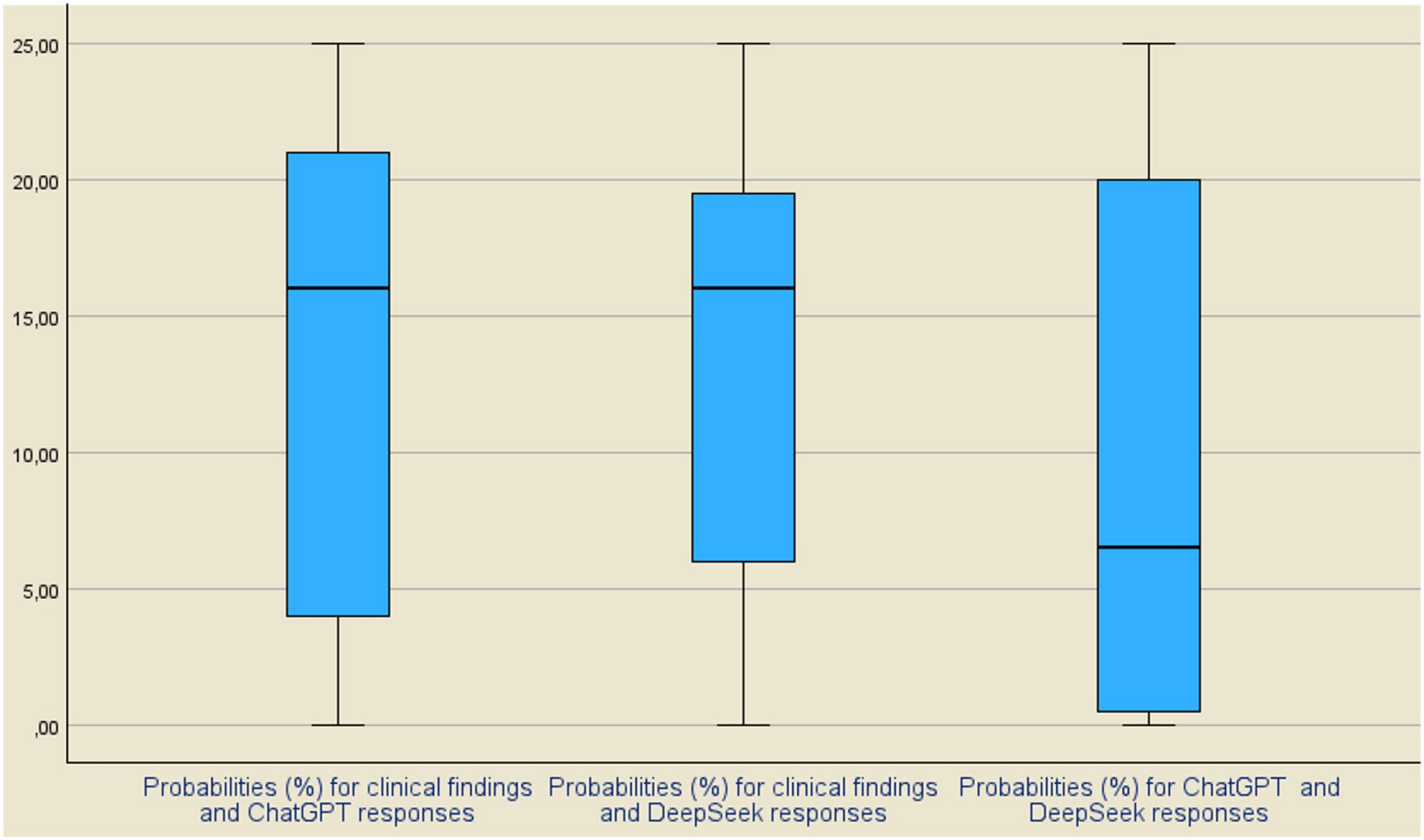
Boxplots for the probabilities (%) corresponding to the differences d of the mean values ​​of the clinical findings and the GPT-5 and DeepSeek-R1 responses. The middle “boxes” representing the middle 50% of the scores show that for the combinations “Clinical findings – GPT-5 responses” and “GPT-5 responses – DeepSeek-R1 responses”, the dispersion is smaller for the combination “Clinical findings – DeepSeek-R1 responses”. The median has high values ​​for the first two combinations and a lower value for the third

Finally, the differences resulting from the calculations of the probabilities for the distances of the mean values ​​d = m-m_i_ in the three combinations mentioned above are shown in the three Boxplots of Fig. 5. In descriptive statistics, a boxplot is a convenient way of graphically displaying five numerical data points from a set of observations: the smallest observation in the first quartile (Q_1_), the median (m) in the third quartile (Q_3_), and the largest observation. The boxplot shows differences between population values. The distances between the different parts of the boxplot help to show the amount of dispersion and skewness of the data. The boxplot itself indicates the range in which the middle 50% of all values lie. Thus, the lower end of the box is the 1st quartile and the upper end is the 3rd quartile.

The middle “boxes” that represent the middle 50% of scores for the groups concerning the combinations “Clinical findings – GPT-5 responses” and “GPT-5 responses – DeepSeek-R1 responses” are comparatively tall. This means that there is a large dispersion of the values of the corresponding probabilities that were calculated. In contrast, the middle box for the combination “Clinical findings – DeepSeek-R1 responses” is comparatively short, which means that the dispersion of the values ​​of the corresponding probabilities calculated is smaller. Finally, with respect to the median (middle quartile), that marks the mid-point of the data and is shown by the line that divides the box into two parts, has high values ​​for the two first combinations and lower value for the third one.

The general conclusion, which arises from the interpretation of the boxplots, is that there is no satisfactory coincidence of the mean values ​​between the clinical findings and the GPT-5 and DeepSeek-R1 responses, while a greater proximity is observed in the mean values ​​of the GPT-5 and DeepSeek-R1 responses. This conclusion is identical to the conclusions that emerged from the previous analyses, according to which there are significant deviations in the results of the three cases examined.

### Evaluation using other statistical metrics

As mentioned above, the use of Cohen and Fleiss’ Kappa indices is more appropriate for categorical data. For this reason, we additionally calculate the following four statistical indices, which can be used for continuous data and will provide additional insight into the accuracy of the predictions of the two models. Specifically, the following indicators will be calculated: Mean Squared Error (MSE), Root Mean Squared Error (RMSE), Mean Absolute Error (MAE) and Mean Absolute Percentage Error (MAPE). Defining the prediction error 𝑒_𝑡_ as the difference between the actual 𝑌_𝑡_ and the predicted value Ŷ_𝑡_, the calculation formulas of the aforementioned statistical indicators for N total observations are as follows [14, 15, 32, 39]:

### (i) Mean squared error (MSE)

7 $$MSE = {1 \over {\rm{N}}}{\sum\limits_{t = 1}^N {({Y_t} - {{\hat Y}_t})} ^2}$$

MSE is a measure of the quality of an estimator. As it is derived from the square of Euclidean distance, it is always a positive value that decreases as the error approaches zero. MSE is the average squared difference between the observed value in a statistical study and the values ​​predicted by a model. When comparing observations with predicted values, it is necessary to square the differences, as some data values ​​will be larger than the prediction and therefore their differences will be positive, while others will be smaller and therefore their differences will be negative. The lower the values ​​of the index, the more reliable the predictions. If the prediction is perfect, the MSE is zero, while as the distance between the data points and the predicted values ​​increases, the MSE also increases.

### (ii) Root mean squared error (RMSE)

8 $$RMSE = \sqrt {MSE} = \sqrt {{1 \over {\rm{N}}}{{\sum\limits_{t = 1}^N {({Y_t} - {{\hat Y}_t})} }^2}}$$

RMSE exhibits the same properties as MSE. Its advantage over MSE is that it is on the same scale as the data, making it easier to understand. This measure emphasizes larger errors over smaller ones, thus providing a more conservative estimate of model accuracy when large errors are particularly undesirable.

### (iii) Mean absolute error (MAE)

9 $$MAE = {1 \over {\rm{N}}}\sum\limits_{t = 1}^N {\left| {{Y_t} - {{\widehat Y}_t}} \right|}$$

MAE is a measure of the average size of the errors in a collection of forecasts, regardless of their direction. It is measured as the mean absolute difference between the predicted and actual values ​​and is used to evaluate the effectiveness of a forecasting model. The smaller the MAE, the better the predictions align with the actual data. An MAE value of 0 means a perfect prediction, but in most cases, achieving such perfection is unlikely.

### (iv) Mean absolute percentage error (MAPE)

10 $$MAPE = {1 \over {\rm{N}}}\sum \> _{t = 1}^N{{\left| {{Y_t} - {{\widehat Y}_t}} \right|} \over {{Y_t}}} \times 100\% $$

MAPE index is expressed as a percentage and is easier to understand, and it allows for comparison of models with data of different scales. Lower MAPE values ​​indicate higher accuracy, while higher values ​​indicate lower accuracy. A MAPE value of 5% indicates that, on average, the forecasts are 5% off the true values.

The results of the calculations of these statistical metrics are shown in Table 4. As mentioned above, in order for there to be a satisfactory coincidence of the values ​​obtained from the clinical findings and from the responses of the two LLMs, the values ​​of the indicators included in Table 4 should approach zero. However, the results of these calculations gave values ​​that are much greater than zero and therefore confirm the conclusions previously formulated about an unsatisfactory coincidence of the values ​​for the three cases examined.

**Table 4:** Values of other statistical metrics given by formulas (7) to (10)

	Clinical findings-ChatGpt responses	Clinical findings - DeepSeek responses	Clinical findings - DeepSeek responses
MSE	277,40	364,79	257,61
RMSE	16,65	19,09	16,05
MAE	289,3	349,1	238,8
MAPE	39,66	67,56	48,177

The Table includes the indicators: Mean Squared Error (MSE), Root Mean Squared Error (RMSE), Mean Absolute Error (MAE) and Mean Absolute Percentage Error (MAPE), which estimated using the values obtained from the clinical findings and the responses of the two LLMs

## Discussion

In this article, a structured comparative evaluation of two leading LLMs models, ChatGPT (GPT-5) and DeepSeek (DeepSeek-R1), has been carried out, regarding their potential for use in the management of two rheumatological diseases, axSpA and PsA. The evaluation was carried out by comparing the responses obtained from the two models with the clinical data obtained from 116 patients treated at a tertiary rheumatology hospital in Germany and calculating their degree of agreement using appropriate indicators. We believe that the agreement between the values ​​of the clinical data and the responses of the two models is an important indicator of the reliability of the LLMs and their potential for use in the management of rheumatological diseases.

The results obtained by applying appropriate statistical measures showed moderate to satisfactory overall agreement. Specifically, comparisons of mean values ​​showed variable agreement, since in some cases the coincidence of values ​​is very good, in other cases it is satisfactory, while in the rest large differences emerged. Similar are the results obtained from the calculations of the Cohen Kappa and Fleiss’ Kappa coefficients. The results of the calculations of the Fleiss Kappa coefficient do not show satisfactory agreement in the values ​​of the clinical results with the responses of the two models, while the degree of agreement for these categories is not statistically significant. In other words, both Cohen and Fleiss Kappa coefficients consistently showed low to moderate agreement between the results of the LLM and the clinical findings, which leads to the conclusion that the reliability of the two models is limited and their use should be made after criticism by rheumatologists.

Regarding the results that determine the estimation of the confidence level corresponding to the differences in the estimates of the means, these showed that they do not guarantee a satisfactory coincidence of the predictions obtained using the GPT-5 and DeepSeek-R1 models with the clinical results. Also, the general conclusion, which results from the interpretation of the boxplots drawn with the probability data (%) corresponding to the differences d of the values ​​of the clinical findings and the responses obtained by the two models, is identical to the two models. previous conclusions. That is, the existence of significant differences in the mean values ​​of the clinical findings and the responses of the two models is confirmed. Finally, the results obtained from the calculation of the statistical indicators MSE, RMSE, MAE and MAPE, gave values ​​that are much greater than zero, which is a confirmation of the previous conclusions, that there is no satisfactory coincidence of the values ​​for the three cases examined.

The lack of agreement between the clinical findings and the responses of the two models leads to clear views for clinical practice. The quantitative discrepancy documented in this article suggests the impossibility of considering the two models as reliable and independent diagnostic or therapeutic “advisors” in rheumatology, despite the production in a short time of reasonable and well-written responses that superficially resemble those of experts. The consistently low values ​​of agreement between clinical data and responses of the two models indicated by the statistical indices calculated, highlight the lack of satisfactory diagnostic accuracy of the LLMs. This quantitative approach can be used as a basis for future comparative evaluation studies of the LLMs. The use of multiple statistical measures, such as mean differences with confidence intervals, Cohen’s Kappa (pairwise) and Fleiss’s Kappa (multiple assessment), and other indices, offers a more robust framework than simple quota agreement because it captures the agreement that occurs by chance.

We therefore believe that at this stage of development, LLMs should be considered exclusively as complementary tools for the management of rheumatological or other diseases. They may be useful for patient education, the creation of differential diagnosis lists for medical evaluation, the writing of plain language explanations, or the support of bibliographic searches, but never as a substitute for evaluation by specialist physicians.

The creation of LLMs that have the ability to analyze various types of medical information beyond the formulation of plain text may be the subject of future research. The continuous exploration and improvement of these technologies may lead to the reduction of errors and their successful integration into daily medical practice, with significant benefits for medical and nursing staff, as well as patients.

### Limitations

There are some limitations that should be acknowledged in this study. First, the study is concerned with the use of ChatGPT and DeepSeek in providing information to patients with axSpA and PsA. It is possible that the performance of the two LLMs in other rheumatic diseases (e.g., rheumatoid arthritis, systemic lupus erythematosus, or vasculitis) may be different. Second, it is possible that other LLM models available internationally may provide more reliable information or that future versions of ChatGPT and DeepSeek may be more reliable. Third, the prompts were standardized and relatively simplified compared to the repetitive, context-rich dialogue that occurs in real clinical encounters. More sophisticated prompting or chain-of-thought engineering strategies could improve the performance of the LLM. Finally, the analysis was based on retrospective clinical data rather than prospective real-time applications, so dynamic aspects such as follow-up questions or incorporation of new laboratory/imaging results could not be assessed.

## Conclusions

Based on the results of our analysis, the conclusions are that the responses of the GPT-5 and DeepSeek-R1 models have moderate validity, reliability, and usefulness in providing information to patients with axSpA and PsA. The quantitative estimates of these models do not show satisfactory agreement with the corresponding clinical estimates made by the experts.

In general, the evaluation and benchmarking of LLMs is necessary to ensure the reliability of the responses and the ability of LLMs to handle real-world tasks and provide accurate and applicable answers. Issues related to the use of LLMs, such as the precise and correct formulation of the question by the user, so that it can be understood and interpreted accurately by the model, have not been adequately resolved. This may be due to the different answers given by LLMs after a small variation in the formulation of the question. It is also worth noting that a different answer may be obtained to the same question when it is given for response at different times in the LLMs.

Therefore, the use of these models cannot be done without prior verification by physicians. Their use is not reliable if we do not know their limitations, including the possibility of incomplete or incorrect responses and outdated information. The information obtained from these models can be used after relevant evaluation and validation by physicians. In other words, the safe integration of ChatGPT and DeepSeek into patient care requires that recommendations be cross-referenced with updated clinical guidelines. Otherwise, risks to patient health arise.

Despite these disadvantages, the ChatGPT and DeepSeek models have advantages related to their ability to directly answer questions related to medical conditions and the ease of obtaining information from any person without a medical background. Their use should be complementary and function as an additional tool in the process of providing medical care, while the final decision should always be made by healthcare professionals, since a medical error can be fatal for the patient.

The responses of the GPT-5 and DeepSeek-R1 models should be used as a complementary tool for medical diagnosis and not as a substitute for professional medical advice, diagnosis, or treatment. Despite its multiple and beneficial applications, LLMs cannot, nor should they, replace specialized medical advice, diagnosis, or treatment. Their responsible use, complemented by professional medical expertise, can make them valuable tools for medical science.

Finally, it is concluded that further studies with longer evaluation periods and larger samples are needed to gain a more comprehensive understanding of the performance of LLMs in the diagnosis of rheumatologic diseases and to effectively utilize the potential of AI technology in rheumatology. While ChatGPT and DeepSeek demonstrate remarkable language capabilities and accessibility, their current diagnostic and treatment recommendations in axSpA and PsA exhibit only moderate validity and reliability when measured against expert clinical judgment. Safe integration into rheumatology workflows will require continued technical advancement, domain-specific improvements, transparent reporting of training data and limitations, and—most importantly—rigorous, quantitative validation studies of the kind presented here. Until such evidence accumulates, these tools should complement, and never replace, expert expertise.

## Supplementary Information

Below is the link to the electronic supplementary material.

Supplementary Material 1

## Data Availability

The research was conducted in the context of the corresponding author’s ongoing doctoral dissertation and the data are available from her upon reasonable request.
