# Supplementary material for: Artificial Intelligence (AI) in rheumatology: a comparative evaluation of the ChatGPT and DeepSeek application
Source: BMC Rheumatol. 2026 Feb 12;10:13. doi: 10.1186/s41927-026-00618-y (PMC12896147; doi:10.1186/s41927-026-00618-y)
Supplement: Supplementary file 1 — Supplementary Material 1 [file 41927_2026_618_MOESM1_ESM.docx]

**Artificial Intelligence (AI) in Rheumatology: A comparative evaluation of the ChatGPT and DeepSeek application**

**Maria Polyzou^1,2^*, Xenofon Baraliakos^2^**

**^1^**Department of Pathophysiology, School of Medicine, National and Kapodistrian University of Athens, Laiko General Hospital, Athens, Greece

**^2^**Ruhr-University Bochum, Rheumazentrum Ruhrgebiet, Ruhr-University Bochum, Herne, Germany

*****Corresponding author: Maria Polyzou, email: [mary_polyzou@hotmail.com](mailto:mary_polyzou@hotmail.com)

**APPENDIX**

**Table A-1: Questions given to answer in the ChatGPT and DeepSeek models**

**Prompt:**

Please answer one-by-one the following questions including information of the exact model version and the inference parameters (temperature, top-p, number of runs) you used:

| **No** | **Question** |
| --- | --- |
| 1 | What is the percentage of patients in the Rhineland-Westphalia region of Germany aged 26 to 72 years, who suffer from axSpA and present at the time of diagnosis with morning stiffness in the spine? |
| 2 | What is the percentage of patients in the Rhineland-Westphalia region of Germany, aged 26 to 72 years, who suffer from PsA and present at the time of diagnosis with morning stiffness in the spine? |
| 3 | What percentage of patients in the Rhineland-Westphalia region of Germany, aged 26 to 72 years with axSpA, have a history of psoriasis? |
| 4 | What percentage of PsA patients in the Rhineland-Westphalia region of Germany, aged 26 to 72 years have psoriasis? |
| 5 | What percentage of patients in the Rhineland-Westphalia region of Germany, aged 26 to 72 years with axSpA have pain improvement with the use of NSAIDs? |
| 6 | How many patients in the Rhineland-Westphalia region of Germany, aged 26 to 72 years with PsA have pain improvement with the use of NSAIDs? |
| 7 | At what age does axSpA occur in the Rhineland-Westphalia region of Germany? |
| 8 | At what age does PsA occur in the Rhineland-Westphalia region of Germany? |
| 9 | What is the male/female ratio in axSpA in the Rhineland-Westphalia region of Germany in patients aged 26 to 72 years? |
| 10 | What is the male/female ratio in PsA in the Rhineland-Westphalia region of Germany in patients aged 26 to 72 years? |
| 11 | What is the percentage that the layperson's possible diagnosis of axSpA in the Rhineland-Westphalia region of Germany in patients aged 26 to 72 years, coincides with the specialist's diagnosis? |
| 12 | What is the percentage that the lay doctor's possible diagnosis of PsA in the Rhineland-Westphalia region of Germany in patients aged 26 to 72 years, coincides with the specialist's diagnosis? |
| 13 | What percentage of patients aged 26 to 72 years in the Rhineland-Westphalia region of Germany are HLAB27 positive in axSpA? |
| 14 | What percentage of patients aged 26 to 72 years in the Rhineland-Westphalia region of Germany are HLAB27 positive in PsA? |
| 15 | What percentage of patients aged 26 to 72 years in the Rhineland-Westphalia region of Germany, who are smokers develop axSpA? |
| 16 | What is the percentage of patients aged 26 to 72 years in the Rhineland-Westphalia region of Germany, who are smokers and develop PsA? |
| 17 | What is the percentage of patients aged 26 to 72 years in the Rhineland-Westphalia region of Germany, who have a positive MRI for sacroiliitis at the time of diagnosis of axSpA? |
| 18 | What is the percentage of patients aged 26 to 72 years in the Rhineland-Westphalia region of Germany, who have a positive MRI for sacroiliitis at the time of PsA diagnosis? |
| 19 | What percentage of patients aged 26 to 72 years in the Rhineland-Westphalia region of Germany with axSpA develop uveitis? |
| 20 | What percentage of PsA patients aged 26 to 72 years in the Rhineland-Westphalia region of Germany develop uveitis? |
| 21 | What percentage of patients with axSpA aged 26 to 72 years in the Rhineland-Westphalia region of Germany experience diarrhea? |
| 22 | What percentage of PsA patients aged 26 to 72 years in the Rhineland-Westphalia region of Germany experience diarrhea? |
| 23 | What percentage of patients with axSpA aged 26 to 72 years in the Rhineland-Westphalia region of Germany experience dactylitis? |
| 24 | What percentage of PsA patients aged 26 to 72 years in the Rhineland-Westphalia region of Germany experience digitalis? |
| 25 | What percentage of patients with axSpA aged 26 to 72 years in the Rhineland-Westphalia region of Germany have limited range of motion at the time of diagnosis? |
| 26 | What percentage of patients with PsA aged 26 to 72 years in the Rhineland-Westphalia region of Germany have limited range of motion at the time of diagnosis? |
| 27 | What percentage of patients with axSpA aged 26 to 72 years in the Rhineland-Westphalia region of Germany have elevated CRP? |
| 28 | What percentage of PsA patients aged 26 to 72 years in the Rhineland-Westphalia region of Germany have elevated CRP? |

**Table A-2: ChatGPT response results and clinical findings**

**Model: GPT-5**

**Parameter - Typical Estimated Range - Purpose**

**Temperature:** ~0.2 – 0.4 - keeps wording stable and fact-oriented

**Top-p:** ~0.9 - allows modest sampling diversity while maintaining coherence

**Number of runs: (completions)** 1 - single deterministic completion, no multi-sampling

| **No** | **Responses** | **Clinical findings ** |
| --- | --- | --- |
| 1 | Large international axSpA surveys/cohorts report **very high rates of reported morning stiffness** (e.g., IMAS reports ~93.8% reporting some morning-stiffness severity). Use this as an approximate expectation for German cohorts, but it is **not** NRW+26–72 specific. | 83,3% |
| 2 | Only a subset of PsA patients have axial involvement; axial PsA prevalence varies widely (reported 5–70% depending on definition). Morning spinal stiffness will therefore be appreciably **lower** than in axSpA and occurs mainly in the subgroup with axial PsA. Expect a **substantially lower** percentage than axSpA (range depends on how “axial” is defined). Representative review: axial involvement 25–70% in established PsA; many axial-PsA patients do **not** report classic inflammatory back pain. | 45,8% |
| 3 | In axSpA cohorts, coexistent psoriasis is reported but varies by cohort; family-history/personal-history of psoriasis occurs in a minority (exact varies between 10-15%). Many axSpA/AS cohorts report psoriasis in a **single-digit to low-teens percent range**, but this is cohort-dependent. Use national/regional registry data for precise numbers if available locally. | 12,5% |
| 4 | **By definition nearly all PsA patients have (or had) psoriasis** — PsA is the arthritis associated with psoriasis. Most PsA cohorts report that the **majority (>85–95%)** have current or prior documented psoriasis (timing and severity can vary). | 82,1% |
| 5 | NSAID response rates vary by outcome definition — many trials/observational studies show **any clinically meaningful pain improvement in roughly ~25–60%** depending on measure and follow-up (example: some trials report ASAS/clinical response rates around ~30–50% early; other studies report moderate response rates up to ~57% for specific NSAIDs/definitions). Expect **substantial but not universal** symptom improvement with NSAIDs. | 83,3% |
| 6 | In PsA, NSAIDs are commonly used and often provide symptomatic relief for peripheral/axial pain, but formal responder percentages in PsA trials vs axSpA are variable (~60–70%). Expect symptom improvement in a proportion similar to other inflammatory arthritides; precise % depends on outcome definitions and whether axial disease is present. | 100% |
| 7 | General/typical: axSpA commonly begins in **young adulthood**; classification criteria often use onset **<45 years** for inflammatory back pain suggestive of axSpA. Mean age at diagnosis in many cohorts is in the **30s–40s**. Use national registries (e.g., RABBIT-SpA, RHADAR) for German age distributions if you need exact NRW counts. | 34 |
| 8 | General: PsA typically presents in **adulthood**, most commonly between **30 and 50 years** (but onset can be earlier or later). Population projections and German prevalence studies present age profiles showing increased prevalence in mid-life. | 43 |
| 9 | General: axSpA historically shows male predominance in radiographic disease (AS) but more balanced sex ratios in non-radiographic axSpA; many modern cohorts show **male:female roughly 1.2–2.0:1** depending on the subset. Exact ratio varies by cohort and by whether you count radiographic vs non-radiographic axSpA. | male/female 1:1  that is 50% male |
| 10 | General: PsA usually shows a near **sex-balanced** distribution (male:female ≈ **1:1** or slightly male- or female-predominant depending on the cohort and age group). | male/female  1,0:1,7  that is 37% male |
| 11 | I found no peer-reviewed estimates for “layperson’s” suspected axSpA vs rheumatologist diagnosis rates in NRW and the 26–72 age stratum. Studies do exist on **screening tool performance** (e.g., how inflammatory back pain questions perform) but not “layperson vs specialist” concordance at the regional level. An estimate is about 15%. For screening performance see reviews of inflammatory back pain criteria. | 50,0% |
| 12 | There are studies of primary-care referral accuracy and screening, but not the exact metric you asked at the NRW + 26–72 granularity. A very rough estimate is 30-45%. For general referral/diagnostic delay information, see RABBIT-SpA and IMAS literature. | 48,2% |
| 13 | HLA-B27 positivity in axSpA varies by population and study: reported ranges are roughly **~60% to >90%** (higher in radiographic AS, lower in non-radiographic axSpA). A recent review notes global variation; many European cohorts have HLA-B27 prevalences toward the higher end (e.g., 80%+ in many classic AS cohorts). Use cohort-specific reports for exact NRW numbers. | 54,1% |
| 14 | HLA-B27 positivity in PsA cohorts is **much lower** than in axSpA, commonly reported around **~15–25%** (values of ~20% are frequently cited). HLA-B27 is more common among the PsA patients who have axial involvement. | 14,2% |
| 15 | What is known: smoking is associated with worse axSpA disease activity, more radiographic progression and poorer outcomes. Quantifying “what % of smokers develop axSpA” requires cohort incidence data and population smoking prevalence; I didn’t find a public NRW-level incidence by smoking status. For risk associations see cohort analyses and reviews. | 50,0% |
| 16 | What is known: smoking is a recognized environmental risk factor that influences psoriasis and PsA risk and course, but precise absolute risk (% of smokers who go on to develop PsA) is not reported at NRW + 26–72 granularity in public literature. Population-level risk estimates require large longitudinal data; see psoriasis→PsA risk studies for approximate incidence ranges. | 35,0% |
| 17 | In axSpA, MRI sacroiliitis (bone marrow edema etc.) is a core diagnostic feature and is present in a substantial fraction of patients at diagnosis; exact percentages vary by cohort and by whether radiographic changes already exist. In early axSpA cohorts many patients show MRI sacroiliitis (proportions vary widely — some early cohorts report 30–70% depending on selection). Use regional registry imaging subanalyses for an exact local figure. | 66,6% |
| 18 | MRI-documented sacroiliitis occurs in the subgroup of PsA patients with axial involvement; because axial PsA prevalence varies, the fraction with positive MRI will be a subset (varies widely; studies show axial PsA detection by MRI in cohorts but no NRW-specific rate). Use a very rough estimate **~**20-35%. | 7,1% |
| 19 | Anterior uveitis (acute anterior uveitis) occurs in axSpA/AS with a **population prevalence around ~20–33%** across many studies (meta-analyses report ~25–33% lifetime prevalence of uveitis among SpA patients). So expect roughly **~20–33%** in many axSpA cohorts (not NRW-specific). | 12,5% |
| 20 | Uveitis prevalence in PsA cohorts is **lower than in axSpA**, commonly reported in many studies at **~5% (range ~2–18% depending on cohort)**. Recent single-center and cohort studies report estimates ~4–7% in many PsA samples. | 7,1% |
| 21 | Diarrhea per se is usually a symptom of concurrent IBD. Clinical IBD in axSpA cohorts is reported roughly **5–10%**; subclinical gut inflammation is much higher (up to ~40–50% in some studies). So expect **~5–10%** clinical IBD (diarrhea as a symptom) in many axSpA samples, with higher rates of subclinical intestinal inflammation. | 4,1% |
| 22 | Clinical IBD among PsA patients is less common than in axSpA but elevated compared with the general population; incidence/prevalence in PsA cohorts is variable (often ~1–5% for clinically manifest IBD, cohort dependent). Diarrhea as a symptom can also be medication-related or coincident GI disease; exact % depends on cohort and definitions. | 3,5% |
| 23 | Dactylitis is a hallmark of PsA and is **uncommon in pure axSpA**. Most axSpA cohorts report dactylitis rarely (single-digit percentages). If a patient has concomitant peripheral SpA features, dactylitis may be more common. A rate of approximately **~**8-14% has been found. | 8,0% |
| 24 | In PsA cohorts dactylitis is common — many series report **~30–50%** lifetime prevalence of dactylitis (some reviews cite ~40%). Use that range cautiously for Germany; cohort definitions differ. | 28,5% |
| 25 | Reduced spinal mobility is frequent in axSpA; many cohorts document measurable reductions in metrology indices at diagnosis. Exact % with clinically “limited ROM” depends on the definition used — objective tests and BASMI/metrology data are used in cohorts. A rate of approximately **~10**-16% has been found. | 25,0% |
| 26 | Range of motion limitations occur when axial involvement or severe peripheral joint damage exists; prevalence varies by cohort. A rate 10-18% has been found in public literature. | 14,2% |
| 27 | Across axSpA cohorts, **CRP elevation at baseline** is variable; many studies report **~30–50%** with elevated CRP at presentation (some lower, some higher). Elevated CRP is common but not universal. | 41,6% |
| 28 | In PsA cohorts, an elevated CRP is present in a **substantial minority** (several reports cite ~30–40% depending on disease activity and cohort). Note that many PsA patients have normal CRP despite active disease. | 17,8% |

Note: All answers are marked "*There are no published data specifically for Rhineland-Westphalia limited to ages 26-72*".

**Table A-3: DeepSeek response results**

**Model and Inference Parameters:**

- **Model Version:** **DeepSeek-R1**
- **Temperature:** 0.3 (Selected for more deterministic and focused outputs)
- **Top-p:** 0.7
- **Number of Runs:** 1

| **No** | **Responses** |
| --- | --- |
| 1 | An estimated **70-90%** of patients with axSpA report morning stiffness in the spine at the time of diagnosis. This is a core clinical feature of the disease. |
| 2 | An estimated **50-70%** of patients with PsA present with morning stiffness. However, in PsA, stiffness is often more prominent in peripheral joints than in the spine, unless axial involvement is present. |
| 3 | An estimated **10-25%** of patients with axSpA have a co-existing diagnosis of psoriasis. |
| 4 | By definition, PsA occurs in the context of psoriasis. An estimated **85-100%** of PsA patients have a current personal history of psoriasis or will develop it. In a small minority, arthritis can precede the skin manifestations. |
| 5 | A good to excellent response to NSAIDs is a key diagnostic feature. An estimated **70-90%** of axSpA patients experience significant pain and stiffness improvement with NSAIDs. |
| 6 | This question asks for a number, but without a total patient population, a percentage is more meaningful. An estimated **50-70%** of PsA patients experience pain improvement with NSAIDs, though they are often insufficient as monotherapy. |
| 7 | axSpA typically has a symptom onset in the **late teens to late 20s**. The median age of onset is often cited as around **26 years**. |
| 8 | PsA typically develops between the ages of **30 and 50 years**, with a peak onset around **40 years**. |
| 9 | Historically, axSpA was thought to be male-predominant. More recent data suggests the ratio is closer to **~2:1 to 1:1 (male:female)**, especially when non-radiographic axSpA is included, which is more common in women. |
| 10 | PsA affects men and women nearly equally, with a ratio of approximately **1:1**. |
| 11 | A layperson's (non-medical) guess would have a very low concordance rate. Based on the complexity of the diagnosis, the estimated percentage is **<10%**. |
| 12 | A "lay doctor" (e.g., general practitioner) would have a better chance than a non-medical person. The concordance rate with a rheumatologist's diagnosis is estimated to be **~30-50%**, as PsA can mimic other forms of arthritis. |
| 13 | The prevalence of HLA-B27 is high in axSpA. An estimated **80-90%** of patients with radiographic axSpA (Ankylosing Spondylitis) are positive, and **50-70%** of those with non-radiographic axSpA are positive. |
| 14 | HLA-B27 is less common in PsA than in axSpA. An estimated **10-25%** of all PsA patients are positive. This percentage is higher in PsA patients with axial involvement. |
| 15 | This question is phrased backwards. A more standard epidemiological measure is the risk associated with smoking. It is not possible to state what percentage of smokers develop axSpA. However, smoking is a known strong environmental risk factor for developing axSpA and is associated with worse disease severity. |
| 16 | Similar to the previous question, a direct percentage is not standard. Smoking is a risk factor for psoriasis and may be associated with a higher risk of developing PsA, but a specific percentage for the development of PsA among smokers cannot be reliably estimated here. |
| 17 | Active inflammatory sacroiliitis on MRI is a key diagnostic criterion. An estimated **60-80%** of patients with axSpA will have a positive MRI for sacroiliitis at diagnosis. |
| 18 | Axial involvement occurs in a subset of PsA patients. An estimated **25-40%** of PsA patients have a positive MRI for sacroiliitis. |
| 19 | Acute anterior uveitis is a common extra-articular manifestation. An estimated **25-40%** of axSpA patients will experience at least one episode of uveitis. |
| 20 | Uveitis is less common in PsA than in axSpA. An estimated **7-15%** of PsA patients develop uveitis. |
| 21 | Diarrhea is not a direct symptom of axSpA but can occur due to associated Inflammatory Bowel Disease (IBD) or NSAID use. An estimated **5-10%** of axSpA patients may experience chronic diarrhea, primarily linked to these comorbidities. |
| 22 | Similar to axSpA, diarrhea is not a primary feature. An estimated **<5%** of PsA patients experience diarrhea directly related to their condition, though it can also be a side effect of medications. |
| 23 | Dactylitis ("sausage digits") can occur in axSpA but is more characteristic of PsA. An estimated **5-15%** of axSpA patients experience dactylitis. |
| 24 | I assume this is a typo for "dactylitis." Dactylitis is a hallmark feature of PsA. An estimated **30-50%** of PsA patients experience dactylitis. |
| 25 | Limited spinal mobility is a core feature. An estimated **60-80%** of patients have some degree of objectively measured limited range of motion in the spine at diagnosis. |
| 26 | Limited range of motion in PsA is common in affected joints. An estimated **50-70%** of patients have limited range of motion in peripheral or axial joints at diagnosis. |
| 27 | CRP is elevated in a significant proportion, but not all, patients. An estimated **50-70%** of axSpA patients have an elevated CRP at diagnosis, especially those with more active inflammatory disease. |
| 28 | CRP elevation is common but not universal. An estimated **40-60%** of PsA patients have an elevated CRP at diagnosis. |
